# Experiences of Ghanaian frontline healthcare workers during the COVID-19 pandemic and healthcare leadership recommendations

**DOI:** 10.1371/journal.pgph.0004992

**Published:** 2025-07-24

**Authors:** Kimesha Grant, Holly Sims, Alexis Carbine, Jessica Walczak, Yvonne Commodore-Mensah, Thomas Hinneh, Fred Stephen Sarfo, Lisa A. Cooper, Carmen Alvarez

**Affiliations:** 1 Johns Hopkins University School of Nursing, Baltimore, Maryland, United States of America; 2 Johns Hopkins Center for Health Equity, Baltimore, Maryland, United States of America; 3 Johns Hopkins University School of Medicine, Department of Medicine, Division of General Internal Medicine, Baltimore, Maryland, United States of America; 4 Johns Hopkins Bloomberg School of Public Health, Department of Health, Behavior and Society, Baltimore, Maryland, United States of America; 5 Kwame Nkrumah University of Science and Technology, Kumasi, Ghana; 6 University of Pennsylvania, School of Nursing, Philadelphia, Pennsylvania, United States of America; Griffith University, AUSTRALIA

## Abstract

Understanding the experiences of frontline healthcare workers across different national and resource contexts is important for learning how to best support these providers to optimize their services during extended health emergencies. Using qualitative methods, we conducted 20 semi-structured interviews with frontline nurses and community healthcare workers to understand their working conditions, challenges, and supports during the COVID-19 pandemic. Ghana was selected as a leader in healthcare reform among African nations. Thematic analysis revealed the following themes: challenges and stressors, government support, overcoming challenges, and recommendations for leadership in healthcare organizations. Overall, healthcare workers experienced a plethora of challenges both at work and in their personal lives, including decreased access to food and medical care. They also contended with limited personal protective equipment and higher patient volumes. Despite these challenges, support from employers and the government remained limited. Participants provided several recommendations for healthcare systems and leadership to better support their workforce.

## Introduction

In the last few decades, Ghana has emerged as a leader among African nations in healthcare reform and in response to major health crises. In 2003 with its launch of the National Health Insurance Scheme (NHIS), Ghana was one of the first countries in Africa to implement a universal health care system [1]. This NHIS has been recognized for its success in expanding access and affordability of health care, particularly among lower-income individuals [1]. Ghana’s response to the COVID-19 pandemic was praised as one of the most effective in the continent [2], serving as a model by which other African nations may use in developing their responses to national or global crises. Understanding both the successes and areas for growth of the Ghanaian healthcare response to the pandemic is warranted to provide lessons and recommendations that may be used in future crises.

Frontline healthcare workers (HCWs) were instrumental in the immediate and ongoing responses to the COVID-19 pandemic. Healthcare workers practicing during the peak of the pandemic experienced disruptions in their home and working conditions, leading to increased rates of burnout, stress, and other poor health outcomes [3–5]. Tasked with higher patient volumes and acuities, strict hygiene and social distancing protocols, and increased job-related demands, HCWs experienced unique stressors during the pandemic and potential exacerbations of job-related burnout [6,7]. For frontline HCWs employed in settings already struggling with limited resources, the pandemic may have further decreased access and availability of supplies and support for HCWs to effectively function in their roles.

Even though disruptions in daily life and negative mental health effects have been found among HCWs globally [6,8], it is important to consider that HCWs’ experiences vary based on country and preexisting support systems. In the United States, frontline HCWs experienced stressors related to high volumes of COVID-19 patients, limited capacity for non-COVID cases, and mandatory increased working hours to accommodate the unprecedented influx of cases [7]. Ghanaian frontline HCWs faced challenges unique to their experiences and perceptions of the COVID pandemic. For example, the majority (62.5%) of HCWs expressed misconceptions about the etiology of COVID-19 and over 40% did not feel adequately prepared to care for COVID-19 patients [9], resulting in increased rates of stress, stigmatization, and burnout related to lack of preparedness and fear of infection [10–12]. To support Ghanaian HCWs, a unique approach is warranted that may differ from the recommendations abundant in the literature of HCWs in the US and other Western nations, in consideration of varying degrees of resource availability and public perception.

Using qualitative descriptive methods, we aimed to gain an understanding of the stressors, challenges, and supports experienced by Ghanaian frontline HCWs during the pandemic. In Ghana, a country that has struggled with resource insufficiency even prior to the pandemic [13], practical support solutions are warranted based on the most pertinent needs of HCWs and patients to whom they provide care. This study fills a gap in the literature by exploring the daily pandemic-related stressors of HCWs in an under-resourced country and includes recommendations for how they can be better supported during health crises.

## Methods

### Study design

Using a qualitative descriptive methodology, we conducted individual, semi-structured interviews to explore the experiences of Ghanaian healthcare workers during the pandemic. Open-ended questions prompted participants to reflect on the stressors they faced at home and work while navigating the changes brought about by the pandemic.

### Recruitment and data collection

This study was conducted in collaboration with a large healthcare system in an urban area of Ghana. Although located in an urban setting, the health system also provides services in rural settings. To recruit a convenience sample of healthcare workers, research partners in Ghana used word-of-mouth and distributed study fliers via email. The email flier contained a hyperlink to REDCap, where prospective participants viewed the consent form. Following the consent form, two questions were included to ensure participants understood that their participation was voluntary. Prospective participants could then indicate their consent to participate and if they wished to participate in the semi-structured interviews. Recruitment was conducted during April and May of 2021. The eligibility criteria included healthcare workers (including but not limited to nurses, community health workers, and social workers) who (1) worked at least 20 hours per week in direct patient care; (2) had been working at their current location for at least a year; and (3) served a predominantly underserved population (e.g., very low-income, rural). We did not include physicians or other primary care providers in the study. Interested participants notified the onsite Ghanaian study team who scheduled interviews and provided transportation to a private location within the health system where participants could engage in remote interviews with the US-based team.

Semi-structured interviews were conducted between April and May 2021 using the Zoom video platform. The interviewers were located in the US and included the study principal investigator and doctoral and masters-level nursing students. This methodological decision was made because this study in Ghana was conceived as part of a larger, ongoing funded project investigating worker experiences during the COVID-19 pandemic across diverse global contexts. Utilizing a single, centralized research team for interviews across all study sites minimized variation in interview approach, procedural protocols, and data collection consistency, thereby enhancing the comparability of findings within the broader project. All interviews were audio-recorded, conducted in English (the official language in Ghana), and lasted 45 minutes on average. Audio files were securely stored on a firewalled, encrypted server, with access restricted to the PI and research coordinator. Participants were compensated an equivalent of US $5 for their time.

#### Consent.

Participants viewed the consent form in REDCap and noted their consent to participate in the study.

#### Ethics statement.

The study protocol was approved by the Johns Hopkins University Institutional Review Board (IRB00252191) and Kwame Nkrumah University of Science and Technology Committee on Human Research, Publication, and Ethics (CHRPE/AP/493/21).

### Analysis

Interviews were transcribed verbatim, de-identified, and uploaded into NVivo12 qualitative data analysis software. After confirming the accuracy of the transcripts using the audio recordings, the audio files were securely deleted. We conducted a thematic analysis following Braun and Clarke’s guidelines [14]. Four study team members independently read the interview transcripts and noted their initial impressions of the data and potential codes. The team collectively discussed the identified codes and created a shared codebook that captured the most emergent patterns from the data. Three iterations of the codebook were performed until the final version of the codebook was agreed upon by all team members. Using the final version of the codebook, each interview transcript was coded independently by two members of the research team, and the data were merged using the coding comparison feature in NVivo12. Areas of discrepancy were reconciled as a group. Relevant codes and interview quotes were matched with each theme to support its inclusion in the final thematic analysis.

## Results

Twenty HCWs (n = 20) participated in the study. Participants were on average 34 years old (range: 24–52), and most were female (n = 14, 70%), married (53%, n = 9), and had children under the age of 18 living in their home (70%, n = 15). To ensure confidentiality, demographic information was not tabulated. Roles and practice settings of the 20 participants are listed in Table 1.

**Table 1:** Healthcare worker roles and practice settings of study participants.

Participant	Role	Setting
1	Nurse	Rural Communities
2	Senior Nurse	Emergency Department
3	Nursing Officer	Outpatient Department
4	Community Health Nurse	Hospital/Clinic
5	Nurse	Hospital
6	Community Health Nurse	Hospital/Clinic
7	Nurse	Specialty Clinic
8	Community Health Nurse	Various Communities
9	Nurse	Emergency Department
10	Community Health Nurse	Various Communities
11	Nurse	Inpatient & Outpatient General Medicine
12	Healthcare Worker	Hospital
13	Nursing Officer	Emergency Department
14	Nursing Officer	Teaching Hospital
15	Nursing Officer	Hospital Emergency Department
16	Nurse	Emergency Department
17	Senior Staff Nurse	Specialty Outpatient Clinic
18	Principal Nursing Officer	Specialty Clinic
19	Midwife	Outpatient Department
20	General Nurse	Male Special Ward

The themes that emerged from the semi-structured interviews were challenges and stressors, governmental support, overcoming challenges, and recommendations. Details are described below.

### Challenges and stressors

#### Fear.

Fear of contracting and spreading the coronavirus was a stressor that transcended both the personal and professional lives of HCWs. Participants explained how lack of information about the virus (e.g., how it was transmitted, treated, and its long-term side effects) contributed to feelings of fear. In describing initial feelings when COVID first emerged, one HCW shared:

*…we were all panicking. We didn’t know how the disease looked like…we didn’t know whether it was airborne....* (*Community Health Nurse, Participant 4*)

These feelings of fear were exacerbated by the lack of PPE in clinical settings. Several HCWs described fear and hesitancy to serve their roles due to unknown COVID statuses of patients entering the hospital.

*...the fact that you are going to take care of a COVID patient, you have your preconceived ideas in mind. Although you are doing what you are being paid to do, but at the back of your mind, you are scared. You are just scared because what if you also contract the disease? (Senior Emergency Department Nurse, Participant 2)*

Even as time progressed and PPE became more readily available, there was still a large sense of fear of contracting the disease and potentially spreading it to loved ones at home. Many respondents coped by taking steps to prevent spreading the virus to their families by isolating at home and going through a cleaning process when returning home from work:

*It was quite stressful because you would come in and then—like you would have to put your shoes out, air-dry, not really knowing even what you were doing was the right thing. How to decontaminate your shoes, how to decontaminate your clothing, it was stressful. (Emergency Department Nurse, Participant 13)*

#### Stressors at home.

Throughout the pandemic, HCWs reported numerous stressors at home. Most prominent was the socioeconomic impact, which restricted access to essential items. Respondents shared challenges with the rising cost of groceries, household goods, and housing costs such as rent and mortgages. One person shared that they took a bank loan to pay the rent. Participants who resided with other family members shared how more expensive goods limited what other family members could contribute to the household, consequently shifting the financial burden to the HCW.

There were also significant disruptors to home life as HCWs changed the ways in which they interacted with their family and friends. Prior to the pandemic, many shared that they went to parties and socialized with friends. For example, one nurse recalled large gatherings on Sundays to watch football (soccer) matches on large screens. Such activities had to stop because of lockdowns which led to a sense of isolation among respondents. Even home gatherings stopped, as another HCW shared:

*We used to have a family meeting. We used to share time together. We used to pray together. We used to do everything together, but when COVID set in, we were not doing such things…. (Hospital Nurse, Participant 5)*

HCWs with children in the home faced increased stressors. Those with school-aged children had to contend with online learning and teaching their children at home. Parents fearful of potentially transmitting the virus to their children described trying to maintain a safe distance from their children until after showering and changing their clothes. When children returned to school, HCWs explained the need to “sanitize” their children’s belongings before bringing them inside the home. For HCWs who did not want their children to eat meals outside of the home (due to concerns about COVID), there was the additional responsibility of preparing to-go meals.

#### Stressors at work.

Participants shared numerous stressors at work arising from the pandemic including PPE shortages, extended work hours, and lack of support from organizational leaders. HCWs in hospital settings explained the tension they experienced with having to care for an increasing number of patients and PPE not being available. HCWs working in community settings also experienced shortages of PPE and reported ultimately having to buy their own:

*They give you one to work with, instead of giving you maybe a pack, and you would be using it every day. You can’t get it. They will not give it to you. We use our own money to buy PPEs for the work. (Community health nurse, Participant 8)*

For HCWs serving rural areas, COVID-19 exacerbated pre-exiting challenges. HCWs routinely conducted well child visits and administered vaccinations without basic equipment (e.g., tables, chairs) to support such activities, and transportation was their responsibility. Once the pandemic began, public transportation became less reliable. In addition, they increasingly served people with very low incomes and exacerbated chronic diseases and other conditions because they were unable to afford or obtain medication.

Other common work-related stressors emerged from compounding responsibilities related to the pandemic: longer working hours, higher acuity patients, limited beds, and difficulty implementing COVID protocols due to patient and staff resistance. Despite enhanced patient triage and treatment referral systems, HCWs could not always rely on the accurate reporting of patients’ symptoms due to social distancing recommendations, thus jeopardizing the utility of such systems.

Some HCWs also experienced interpersonal challenges with colleagues. HCWs discussed experiencing and witnessing hostility and stigmatization when a staff member had an actual or suspected COVID diagnosis. One participant felt so ostracized by the other nurses after having COVID that, even after returning to work with negative results, they still experienced overt differential treatment from colleagues as they explained: “…*when I would sit on a chair...they wouldn’t want to sit on the chair next to that chair…” (Inpatient and Outpatient General Nurse, Participant 11).* This stigmatization ultimately led this person to transfer to another unit for a period of months.

Amidst these challenges, many participants reported receiving little support from their organizational leaders. In general, there was a lack of clear guidance for HCWs on how to systematically navigate new challenges, where and how to seek assistance if the staff member contracts COVID-19, and how to manage increased patient needs.

### Government support

The most support participants received came from government initiatives in the form of tax waivers and instrumental social support. Although these efforts were considered helpful, the consensus among participants was that the initiatives were insufficient to meet HCWs’ significant needs. One HCW shared:

*There should have been some money incentives for us ’cause we were working for long hours. Doing 16 hours was like sacrificing for the hospital. It was only the central government tax waiver that was just the incentives for us. I think that wasn’t enough. We should’ve had some bonuses and other stuff like that.” (Emergency Department Nurse, Participant 16)*

The tax waiver mentioned by HCWs refers to a waiver issued by the federal government that allowed HCWs to not pay taxes for a few months. There were also concerns about issues of equity with the tax waiver which was set at 50% of one’s salary. Some HCWs argued that some providers (such as physicians with higher pay) would get a higher gross benefit, whereas non-HCW essential workers such as housekeeping staff would not receive a benefit even though they were being exposed to the same virus. For these reasons, HCWs argued that all people working in healthcare should have received a more equitable incentive.

Distribution of food and transportation were other forms of government support. Some HCWs verified that food was being distributed, “…*the government was giving people food, at times cooked, and at times raw foods …*.” *(Nursing Officer, Specialty Clinic, Participant 18)*. However, similar to the tax waivers, HCWs raised concerns about how the resources were distributed. Many shared that they were at work when food was distributed so they were unable to access it. The government also funded transportation for HCWs, but the benefit was bound by time and route such that only some HCWs would benefit. For example, rural workers often had to rely on their own transportation because rural bus routes were more restricted or absent.

### Overcoming challenges

Overall, HCWs communicated that relying on their own resilience and family support helped them cope during the pandemic. In the words of one HCW: *“We fought it ourselves” – (Nurse Officer, Participant 15).* Facing lack of support from organizational leadership and inadequate assistance from the government, HCWs overcame many of the challenges using personal coping mechanisms and strategies to accommodate the problems experienced at work and at home.

In response to the lack of PPE, many HCWs purchased their own supplies or found ways to deal with shortages of resources in their workplaces. Some even assisted their patients in obtaining PPE or other supplies, whereas others went as far as providing financial and other resource assistance to patients or programs in need.

Many HCWs discussed using natural resources to strengthen their immunity and prevent COVID-19 infection. Participants discussed consuming more fruits and vegetables and taking steam baths. In responding to how they coped with the loss of taste and smell after caring for a patient suspected of having COVID-19, a nurse explained:

*I resorted to steam bath, taking hot steams and those things. Then my vitamin C, and I think that one helped me.” (Nursing Officer Outpatient Clinic, Participant 3)*

Although family was sometimes a stressor, it was also an important source of social support for managing the multiple challenges that emerged from the pandemic. HCWs relied on their families for emotional support and instrumental support such as help with household chores and assistance with childcare and home schooling.

### Recommendations

When asked about the support they needed, HCWs presented several suggestions for their employers and the government to provide more support during public health emergencies. HCWs suggested that organization leadership provide more resources for HCWs and acknowledge staff for additional work performed during times of crisis. HCWs suggested that employers should not only provide sufficient PPE, but also supplement social needs such as providing transportation for HCWs and offering snacks to those working more than 8-hour shifts. HCWs also wanted reassurance that their leadership was aware of and understood what frontline HCWs were experiencing. They also wanted their hard work to be acknowledged, as one participant shared:

*We want our employer to know that we need more support from our employer, like the PPEs, incremental salary, and other allowances. They should give it to us so that we do our best, as we are doing already. Already we are stretching ourselves for them. We are sacrificing for other humans, so they should also do the same for us, us nurses in Ghana.* (*Community Health Nurse, Participant 8*)

HCWs had similar recommendations for how government could better support frontline workers. HCWs shared that the government should have a direct role in supplying PPE, supplementing salaries in recognition of occupational risks, and ensuring reliable transportation for HCWs. Taken together, HCWs felt that their primary support should come from their organizations and the government. As frontline workers risking their health and that of their families, they felt both these entities have a responsibility to provide a safe work environment with the provision of PPE, support their basic social needs, and provide incentives that reflect the risk they face in their daily roles.

## Discussion

This study makes an important contribution to the literature on the experiences of frontline workers in low-resource settings, particularly those working in West Africa. For HCWs already working in settings with limited resources, the pandemic exacerbated many of the environmental issues and supply shortages that impacted HCWs’ ability to provide patient care and protect themselves from occupational hazards. While other studies have focused on COVID-19 knowledge and practices [15,16], we provide data on the impacts on healthcare workers both in their work and personal life. Our results reinforce the findings of previous studies that experiences of healthcare workers during emergencies are not siloed to the work setting, but spill over into their personal lives [17,18]. Participants in our study shared how they navigated multiple challenges, particularly at work. They shared stressors related to the lack of personal safety due to limited PPE and having to care for patients with unknown COVID-19 status at a time when there was no vaccine or established treatment. Fear was a particularly salient experience among respondents and aligns with previous work which found that most healthcare workers in Ghana reported high perceived risk of contracting COVID-19 in the workplace [13]. These fears were not unfounded as frontline healthcare workers have an increased risk of COVID-19 compared to the general community [4].

Despite numerous challenges, healthcare workers shared receiving limited support by organizational leadership and the government. HCWs received some education and PPE from their employers and little else. In general, HCWs felt demoralized and unsupported by their senior leadership and expressed that additional support should have been provided by their organizations since they were providing essential care at great risk to themselves. The importance of organizational leaders providing a safe work environment was cited by other frontline workers globally as instrumental in their ability to be resilient throughout the pandemic [19–21]. It is important to note that healthcare workers are not monolithic and have diverse needs that need to be tailored to the setting where they work.

Respondents shared some areas that reflected support from the government. This included tax savings for nurses and transportation. These governmental actions reflect a model that can be adopted in other countries during public health emergencies.

The Ghanaian government took several steps to bolster and support the healthcare workforce during the COVID-19 pandemic. They recruited additional workers to fill staffing gaps, waived personal income taxes for all workers, and provided an additional 50% base salary bonus to healthcare workers who cared for COVID-19 patients [22]. In addition, the government committed to protecting workers with the provision of PPE, infection control education, and life insurance coverage [22]. Despite these interventions, the overwhelming consensus among participants was that this support was not received by all HCWs, if at all. These deficits raise the issue of equity and how to distribute resources in a way that considers the diversity of needs and accessibility of the workforce. Some relief, such as food distribution, was offered when health care workers were at work; therefore, they were unable to participate. It is important that interventions be delivered at times when they can be accessed by frontline workers. This will mean providing services, including transportation during nights and weekends, to accommodate 24-hour schedules.

The role of the government in supporting frontline healthcare workers during COVID-19 was a distinct finding in Ghana that was not seen in other studies that investigated frontline healthcare workers’ experiences [17,18,21]. Healthcare workers in this study reinforced that the government should have played a primary role in providing them support so that they can continue serving the community. This common perception may be a function of government ownership of healthcare establishments in Ghana.

We acknowledge a few limitations of this study. First, our participants were a convenience sample of HCWs within one large healthcare system. HCWs from other healthcare organizations and even other urban cities within Ghana may have presented other perspectives. Our data also did not include the perspectives of healthcare leadership charged with supporting the departments represented by our HCW participants. Leadership may have offered a different perspective on the organizational support for employees. Further, although we included our Ghanaian partners in the development of our semi-structured interview guide and in our data analysis, because the research was led by a US-based team there may still be nuances of the Ghanian HCW experience that we were unable to capture. Finally, although our participants shared rich insights into their experiences during the COVID-19 pandemic, these findings are context-specific and should not be extrapolated beyond the parameters of this study.

HCWs recommendations for employers and government align with the US Surgeon General’s “Framework for Mental Health & Well-Being in the Workplace” [23]. The framework presents five evidence-based “essentials” for supporting employee well-being in the workplace. Our participants’ comments align with three of the five: 1) protection from harm—physical and psychological safety; 2) connection and community—fostering collaboration and teamwork; and 3) mattering at work. HCWs were committed to performing their duties and wanted resources that would help them feel safe while working. They also wanted their leadership to demonstrate support and commitment to frontline workers by being physically present and communicating with frontline HCWs. Finally, they wanted recognition for their work. Implementation of these essentials are associated with less workplace injury, greater organization productivity, and improved employee well-being [23].

## Conclusion

Ghanaian frontline HCWs provided essential serves to an array of populations in various communities. Despite the personal challenges they experienced, HCWs persevered to support both their families and patient populations. However, there remains plenty of room for healthcare leadership to support the healthcare workforce to feel safer and more supported as they address the health needs of the public. HCWs offer tangible suggestions for healthcare employers to consider for supporting employees, particularly in times of public health crises.
